# Neurofeedback to improve neurocognitive functioning of children treated for a brain tumor: design of a randomized controlled double-blind trial

**DOI:** 10.1186/1471-2407-12-581

**Published:** 2012-12-06

**Authors:** Marieke A de Ruiter, Antoinette YN Schouten-Van Meeteren, Rosa van Mourik, Tieme WP Janssen, Juliette EM Greidanus, Jaap Oosterlaan, Martha A Grootenhuis

**Affiliations:** 1 Psychosocial Department, Emma Children's Hospital AMC, room A3-241, Meibergdreef 9, Amsterdam 1105 AZ, The Netherlands; 2 Pediatric Oncology, Emma Children’s Hospital AMC, room G8-236, Meibergdreef 9, Amsterdam, 1105 AZ, The Netherlands; 3 VU University Amsterdam, Van der Boechorststraat 1, room 1E-41, Amsterdam, 1081 BT, The Netherlands

**Keywords:** Brain tumor, Child, Survivors, Attention, Memory, Processing speed, Neurocognitive functioning, Intervention, Neurofeedback, Protocol, RCT, Double-blind

## Abstract

**Background:**

Neurotoxicity caused by treatment for a brain tumor is a major cause of neurocognitive decline in survivors. Studies have shown that neurofeedback may enhance neurocognitive functioning. This paper describes the protocol of the PRISMA study, a randomized controlled trial to investigate the efficacy of neurofeedback to improve neurocognitive functioning in children treated for a brain tumor.

**Methods/Design:**

Efficacy of neurofeedback will be compared to placebo training in a randomized controlled double-blind trial. A total of 70 brain tumor survivors in the age range of 8 to 18 years will be recruited. Inclusion also requires caregiver-reported neurocognitive problems and being off treatment for more than two years. A group of 35 healthy siblings will be included as the control group. On the basis of a qEEG patients will be assigned to one of three treatment protocols. Thereafter patients will be randomized to receive either neurofeedback training (n=35) or placebo training (n=35). Neurocognitive tests, and questionnaires administered to the patient, caregivers, and teacher, will be used to evaluate pre- and post-intervention functioning, as well as at 6-month follow-up. Siblings will be administered the same tests and questionnaires once.

**Discussion:**

If neurofeedback proves to be effective for pediatric brain tumor survivors, this can be a valuable addition to the scarce interventions available to improve neurocognitive and psychosocial functioning.

**Trial registration:**

ClinicalTrials.gov NCT00961922.

## Background

As a result of improved treatment, the survival rate of children diagnosed with a brain tumor has increased considerably [1]. As a consequence, neurocognitive long-term effects of the tumor and the treatment are reported more often, including deficits in attention, processing speed, and memory [2-4]. Radiotherapy, chemotherapy, tumor location, and longer time since diagnosis are related to worse neurocognitive functioning [5,6]. A major consequence of these impairments is the decline in ability to acquire new skills and information, which leads to an increasing gap in the development between patients and their peers. This, in turn, has its impact on educational results, vocational success and may compromise social competence and quality of life [7].

Butler and Mulhern have emphasized that interventions should be developed to improve neurocognitive functioning and subsequently improve future perspectives of these children [8]. Interventions that are considered relevant for survivors with cancer-related brain injury are cognitive remediation and pharmacotherapy [9,10]. A cognitive remediation program, using techniques from three disciplines: brain injury rehabilitation, special education and clinical psychology, has been developed and tested by Butler and colleagues [9]. Participants in the randomized controlled trial were 161 survivors of a childhood cancer, whose malignancy and/or treatment involved the central nervous system. The results showed improvements in caregiver reported attention and academic achievement, although the effect sizes were modest. Van ‘t Hooft et al. have investigated the effects of a cognitive training program on neurocognitive function with a randomized controlled trial, enrolling 38 patients with acquired brain injury, including 14 brain tumor survivors [10]. The training program consisted of memory and attention exercises, in combination with cognitive behavioral training. The children in the treatment group showed sustained positive effects on memory and attention functioning until six months after the training, but not on processing speed.

Regarding pharmacotherapy, it has been suggested that survivors of childhood cancer may benefit from stimulant medication as used in the treatment of attention deficit hyperactivity disorder (ADHD). Attention deficits in survivors of brain tumors are likely to improve by methylphenidate. Mulhern and colleagues found improvements of attention in 37 long term survivors of a malignant brain tumor after methylphenidate [11]. In a randomized placebo-controlled double-blinded trial including 32 survivors of a brain tumor (n=25) or acute lymphoblastic leukemia (n=7), Thompson et al. found that methylphenidate led to improved sustained attention [12]. A drawback of pharmacotherapy is the possibility of side effects, e.g. sleep disturbance, weight loss, anxiety, and sadness [13]. Also, this medication does not lead to a sustained effect unless the patient continues the pharmacotherapy.

The limited current available options warrant the search for alternatives. Neurofeedback is a relatively new form of therapy, which has never been investigated in pediatric brain tumor survivors. Neurofeedback is a behavioral intervention that is based on the principles of operant conditioning. During the therapy the patient is presented with real-time feedback on his or her brainwaves, as measured by one or more electrodes on the scalp. The patient is reinforced when the brain produces a certain desired wave. Reinforcement may comprise seeing a movie or hearing music. The desired brain wave is determined by a quantified electro encephalogram (qEEG), which is conducted prior to the training.

The effects of neurofeedback have been discovered serendipitously by Sterman, when cats having received feedback of 12–15 Hz on the motor cortex showed to be less susceptible to epileptic seizures [14]. There is a large body of scientific research documenting the effectiveness of neurofeedback for the treatment of diverse pathological conditions as summarized in comprehensive reviews, including ADHD, traumatic brain injury and schizophrenia [15-19].

Strehl et al. showed that children with ADHD were able to learn to regulate their brain activity by neurofeedback [20]. After training, significant improvements in behavior, attention, and IQ scores were found. All changes proved to be stable at six months follow-up after the end of training. Hodgson et al. conclude in their meta-analysis on nonpharmacological interventions for ADHD that neurofeedback resulted in significant improvements of DSM-IV symptoms of ADHD, neurocognitive functioning and behavior [18]. In a comparative study researchers found that the positive effects of neurofeedback for children with ADHD were superior to a computerized attention training at six months follow up [21]. However, to date there is a lack of published studies that employ a randomized placebo-controlled double-blind design when investigating neurofeedback [22].

Brain tumor survivors differ from ADHD patients, as they have structural brain damage caused by the tumor, surgery, radiotherapy and/or chemotherapy. An indication that neurofeedback might be effective in pediatric brain tumor survivors may be derived from the results of studies into the effects of neurofeedback in patients with traumatic brain injury. A review of Thornton and colleagues [23] describes a total of 44 studies (12 RCT, 16 comparative, 16 correlation) with traumatic brain injury patients reporting improved attention, cognitive flexibility, cognitive performance, and problem solving after neurofeedback, providing strong initial support for the idea that neurofeedback could be used in patients with structural brain damage. Subsequently, Aukema and colleagues conducted a pilot study into the feasibility of neurofeedback on 9 brain tumor survivors in our hospital [24]. This study demonstrated that it was feasible to use neurofeedback with brain tumor survivors. All participants completed the training and were positive about the training they received, as they would recommend it to others. Patients reported decreased subjective fatigue after the training. Also the test results showed that processing speed improved in 6 out of 9 patients. These findings warranted the set up of a larger study into the effectiveness of neurofeedback for pediatric brain tumor survivors.

The current paper describes the protocol of the PRISMA study (pediatric research on improving speed, memory, and attention); a randomized controlled double-blind trial, approved by the medical ethical committee of the Academic Medical Centre in Amsterdam. The primary aim of the PRISMA study is to investigate the efficacy of neurofeedback for improving neurocognitive functioning after treatment for a pediatric brain tumor. Secondary, we hypothesize that subsequent to the expected neurocognitive changes achieved with neurofeedback, children will experience improved psychosocial functioning [25]. Neurocognitive functioning will be investigated by tests administered to the patient. Psychosocial functioning will be measured using patient-reported as well as caregiver and teacher reported questionnaires. Assessments will take place pre and post training, as well as six months post training, in order to examine the long-term effects of the training. Comparing the effects of neurofeedback to placebo feedback will assess efficacy of neurofeedback. Pre training results obtained with the brain tumor survivors will be compared to a control group of healthy siblings, to assess the level of dysfunction on the measures used in this study.

## Methods

### Study design

This study is a randomized placebo-controlled double-blind trial, to investigate whether neurofeedback improves neurocognitive functioning in children who have received treatment for a brain tumor (trial number clinicaltrials.gov NCT00961922). After enrollment, patients will be randomized into two groups: (1a) the experimental group, receiving neurofeedback, and (1b) the placebo group, receiving placebo training. In addition, (2) a control group of healthy siblings is included; this group will not receive any training. If effectiveness of neurofeedback is demonstrated after completion of the study, patients in the placebo group will be given the opportunity to receive neurofeedback.

### Participants

Eligible for inclusion are patients in the Netherlands, aged 8 to 18 years, who finished treatment for a brain tumor at least two years prior to enrolment and who suffer from problems in neurocognitive functioning. Problems in neurocognitive functioning include attention problems, problems with information processing speed and/or memory problems as assessed by caregiver report. Exclusion criteria are premorbid diagnosis of ADHD or ADD, a mental or physical condition that prohibits neurocognitive assessment and insufficient mastery of the Dutch language. Siblings, aged between 8 and 18 years, form the control group.

### Intervention

**Figure 1:**
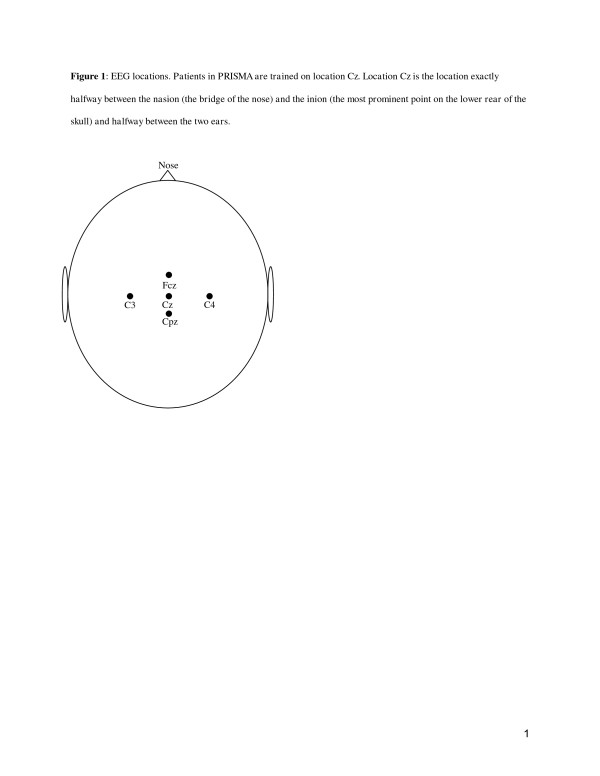
**EEG locations. Patients in PRISMA are trained on location Cz.** Location Cz is the location exactly halfway between the nasion (the bridge of the nose) and the inion (the most prominent point on the lower rear of the skull) and halfway between the two ears.

The neurofeedback training is performed at home or school using a Dell notebook (Inspiron N5030, 15.6 inch screen), with BioExplorer software, version 1.5 installed, and a portable Brainquiry PET neurofeedback device [26,27]. Reinforcement is provided by a self-selected movie that will be presented on the screen if the brain produces the desired activity, as detected by an electrode placed at Cz (see Figure 1). Each patient receives two sessions weekly for 15 weeks, 30 sessions in total. Each session takes 39 minutes to administer, divided in ten blocks of three-minutes training, alternated with one-minute breaks. In the breaks the patient will be instructed to sit quietly with the eyes closed.

The neurofeedback sessions are hosted by extensively trained research assistants who have successfully completed a full day schooling session on administration of the neurofeedback training in accordance with detailed standard operating procedures. During the first neurofeedback session, the research assistant will be accompanied by one of the researchers to ensure adherence to the standard operating procedures. After each session, the research assistant is required to fill out a checklist providing information about the training that includes items on start and finish time of the training, duration of the session, selected movie, alertness of the patient, and any deviations from the standard procedures. Checklists are e-mailed to the researchers on a weekly basis.

#### Neurofeedback treatment modules

The neurofeedback treatment modules were developed in the software program BioExplorer. To increase comparability, we decided to develop three standard treatment modules based on the qEEGs from the pilot study [24], rather than designing an individualized treatment module for each participant. The three treatment modules are (1) beta 1 up training, (2) sensory motor rhythm (SMR) up/beta 1 down training, and (3) beta 1 down training. The qEEG of the patient determines the most suitable of the three neurofeedback treatment modules. The mean Z-score for the power in the beta 1 frequency band (15–20 Hz) for the electrodes on locations Fcz, Cz, C3, C4 and Cpz are calculated (see Figure 1). For SMR no Z-scores are provided in the brain resource report. SMR power is calculated and p-values are obtained over the average of 9 electrodes (F3, Fz, F4, C3, Cz, C4, P3, Pz and P4). The *beta 1 up training* is given if the beta 1 power is within the normal range (within 1 standard deviation from the mean) or lowered (more than 1 standard deviation below the mean). The *SMR up/beta 1 down training* is chosen if the beta 1 power is elevated (more than 1 standard deviation above the mean) and SMR (12–15 Hz) is within the normal range (p>0.05) or lowered (p<0.05). The *beta 1 down training* is chosen if the patient has beta spindles, ascertained by an EEG specialist via observation, or if both beta 1 is elevated (more than 1 standard deviation above the mean) and SMR power is elevated (p<0.05). Three identical placebo treatment modules were matched to the three neurofeedback treatment modules; beta 1 up placebo, SMR up/beta 1 down placebo and beta 1 down placebo. In the placebo treatment modules, the provided reward of a movie is not based on the desired brain waves from the patient, but on the ‘random signal generator’ that is incorporated in the BioExplorer software.

All treatment modules are set at an automatic threshold of 80% reward. This means that the threshold of reward is regularly adjusted in a way that the child sees the movie approximately 80% of the time and 20% of the time the screen of the laptop briefly turns black. Elevated muscular tension and electrical noise from surrounding devices (e.g. a lamp) can decrease the quality of the training; therefore we employ a threshold of 10 μV for muscular tension (> 55 Hz) and 50 μV for noise (range 48–52 Hz). If the muscular tension and/or the noise reach above the threshold, the movie is interrupted and the computer makes a beeping sound. The training will not continue until the muscular tension and/or noise are brought back under the threshold. This applies to the neurofeedback treatment modules as well as the placebo treatment modules.

### Randomization

**Figure 2:**
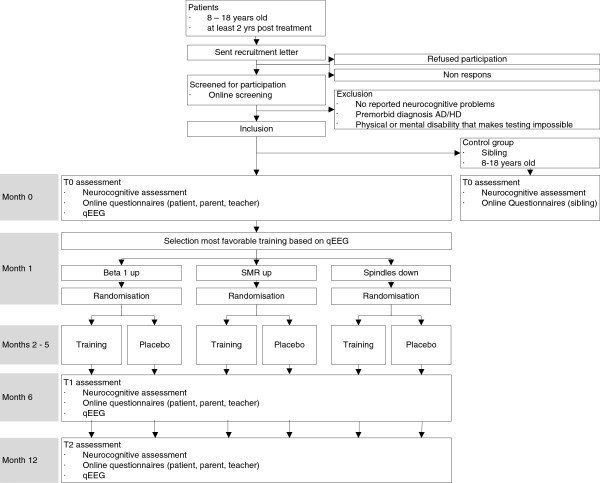
Flow chart of the PRISMA study.

The three neurofeedback treatment modules and three placebo treatment modules have been designed to have the exact same appearance on the screen of the notebook, in order to be indistinguishable during training. The six treatment modules have randomly been assigned a number (treatment module 1–6). J.O., one of the members of the research team, holds the key to the codes; the other members are blinded. Another member of the research team analyzes the qEEG and informs J.O. which of the three neurofeedback treatment modules is indicated according to the protocol (see Figure 2). J.O. is then responsible for randomizing the patient into the actual neurofeedback or the placebo training using a randomization table generated by SPSS. For stratfication purposes, randomization takes place after selection of the most appropriate neurofeedback treatment module. After randomization, J.O. notifies M.d.R. of the assigned treatment module (treatment module 1–6) and M.d.R. sends the assigned treatment module via email to the designated research assistant providing the neurofeedback training. Due to his not-blind status, J.O. is not involved in training any patients or the processing of the data.

### Procedure

#### Recruitment

Five out of seven Dutch University hospitals accepted the invitation to join the study. Participating hospitals are the Emma children’s hospital/Academic Medical Centre in Amsterdam, VU medical centre in Amsterdam, university medical centre Utrecht in Utrecht, St. Radboud university medical centre in Nijmegen and university medical centre Maastricht in Maastricht. A letter via their oncologist or psychologist informs patients and their caregivers about the study. Interested caregivers will be provided with a screening questionnaire concerning their child’s neurocognitive functioning (including items on attention functioning, memory and speed) and exclusion criteria (e.g. premorbid diagnosis of ADHD or ADD) to verify eligibility of the patient. If eligible for inclusion, the patient is invited for the pre training assessment. If applicable, a sibling will also be invited for assessment to participate in the control group. On the day of the pre training assessment, the informed consent form is signed by caregivers, the patient, and if applicable, the sibling.

#### Assessment

Assessments are conducted at one of the three cooperating EEG centers in the Netherlands; Pels institute in Amsterdam, Brainfact in Amsterdam, and EEG resource institute in Nijmegen. Patients are assessed on three occasions: pre training (T0), directly post training (T1), and six months post training (T2); see Figure 2. Assessments include neurocognitive testing, questionnaires filled out by patient, caregiver, and teacher, and a qEEG, see Table 1. Assessment of the siblings occurs only once, and is identical to the assessments used in patients with the exception of questionnaires filled out by parents and teachers.

**Table 1:** Outcomes, measures and to whom it is administered

**Neurocognitive assessment**	**Measurement**	**T0**	**T1**	**T2**
Attention	Attention Network task (ANT) [28]	Patient/sibling (8–18)	Patient (8–18)	Patient (8–18)
Processing speed	Baseline Speed ANT [28]	Patient/sibling (8–18)	Patient (8–18)	Patient (8–18)
Memory	Visual Sequencing task	Patient/sibling (8–18)	Patient (8–18)	Patient (8–18)
	Digit Span (age appropriate Wechsler scale) [29,30]	Patient/sibling (8–18)	Patient (8–18)	Patient (8–18)
Intellectual functioning	Abbreviated WISC-III* [29]	Patient/sibling (8–16)	-	Patient (8–16)
	Abbreviated WAIS-III* [30]	Patient/sibling (17–18)	-	Patient (17–18)
Inhibition	Stop Signal task [31]	Patient/sibling (8–18)	Patient (8–18)	Patient (8–18)
Visuomotor integration	Tracking and Pursuit task	Patient/sibling (8–18)	Patient (8–18)	Patient (8–18)
**Questionnaires**				
Social/emotional functioning	SDQ child version [32]	Patient/sibling (8–18)	Patient (8–18)	Patient (8–18)
	SDQ parent version [32]	Caregiver (8–18)	Caregiver (8–18)	Caregiver (8–18)
	SDQ teacher version [32]	Teacher (8–18)	Teacher (8–18)	Teacher (8–18)
Self esteem	Dutch version SPPC [33]	Patient/sibling (8–11)	Patient (8–11)	Patient (8–11)
	Dutch version SPPA [34]	Patient/sibling (12–18)	Patient (12–18)	Patient (12–18)
Health related quality of life	Kidscreen 27 [35]	Patient/sibling (8–18)	Patient (8–18)	Patient (8–18)
Fatigue	CIS [36]	Patient/sibling (8–18)	Patient (8–18)	Patient (8–18)
Sleep disorder	SDSC [37]	Caregiver (8–18)	Caregiver (8–18)	Caregiver (8–18)
Attention	SWAN [38]	Caregiver (8–18)	Caregiver (8–18)	Caregiver (8–18)
Executive functioning	BRIEF parent version [39]	Caregiver (8–18)	Caregiver (8–18)	Caregiver (8–18)
	BRIEF teacher version [39]	Teacher (8–18)	Teacher (8–18)	Teacher (8–18)

Note: Intellectual functioning is assessed at T0 and T2 but not at T1. Siblings are assessed at T0 only.

*The following subtasks were administered: Arythmic, Similarities, Block Design, and Picture Completion.

SDQ = Strengths and Difficulties Questionnaires; SPPC/SPPA = Self Perception Profile for Children/Adolescents; CIS = Checklist Individual Strength; Sleep Disturbance Scale for Children; SWAN = Strengths and Weaknesses of ADHD-symptoms and Normal-behavior; BRIEF = Behavior Rating Inventory of Executive Functioning; WISC-III = Wechsler Intelligence Scale for Children – Third version; WAIS-III = Wechsler Adult Intelligence Scale – Third version.

##### qEEG

An EEG is recorded from the patients at three time points. A Quick-cap with NuAmp 10–20 electrodes international system from neuroscan is used, with 28 channels [40]; Fp1, Fp2, F7, F3, Fz, F4, F8, FC3, FCz, FC4, T3, C3, Cz, C4, T4, CP3, CPz, CP4, T5, P3, Pz, P4, T6, O1, Oz and O2. During the first three minutes an eyes-open resting EEG is registered, in the consecutive three minutes an eyes-closed resting EEG. After the resting EEG, event related potentials are measured during an odd ball and a go-nogo task. The Brain Resource International Database (BRID) [41,42], comprising EEG power spectra of over 4.000 healthy controls, provides normative data to quantify the EEG (qEEG) and obtain Z-scores for the participants in the current study.

##### Neurocognitive tests

To objectify the primary hypothesis of the study, that neurofeedback will improve neurocognitive functioning, different neurocognitive domains are assessed. The tests are conducted by one of the researchers or extensively trained research assistants and take approximately two and a half hours. Based on literature describing late effects in brain tumor patients, the following neurocognitive domains were targeted for assessment [43]: attention, processing speed, memory, intellectual functioning, inhibition, and visuomotor integration. Well-validated computerized and pencil-and-paper tests were selected to provide a comprehensive assessment of neurocognitive functioning before the training and the efficacy of neurofeedback (see Table 1).

##### Questionnaires

Our secondary hypothesis regards the impact of neuropsychological performance on psychosocial functioning [25,44]. Based on studies reports, the following domains are assessed using questionnaires: social/emotional functioning, self-esteem, and health related quality of life. Because of the reported decrease in fatigue after training in the pilot study, we also included questionnaires assessing fatigue and sleep disturbance [24]. In addition, two questionnaires on attention and executive functioning were added to assess caregiver and teacher rated neurocognitive functioning. Widely used, reliable, and validated questionnaires were selected in order to assess the domains of interest, as well as the effect of neurofeedback on these domains (see Table 1). Questionnaires were administered to either patient, caregivers or teacher, if applicable. The online questionnaires take approximately 30 minutes to fill out. In addition, as an interim measurement, caregivers fill in the attention questionnaire (SWAN [44]) one extra time, after the first 10 sessions of the patient.

### Power calculation

Power calculations used the neurocognitive measures as primary outcome measures. The calculations were done in the statistical program nQuery Advisor [45]. We expect that the neurofeedback will have a medium (d=0.5) to large effect (d=0.8) on neurocognitive functioning as measured at T1 and compared to T0, based on improvements found in children with ADHD who were trained with neurofeedback and on the improvements found in learning-impaired childhood cancer survivors treated with methylphenidate [12,15]. Given an effect size of 0.6 with alpha set at 0.05 (one-sided) and a power of 0.80, a minimum of 35 patients is required in both the neurofeedback group and the placebo group.

### Statistical analyses

Intention-to-treat analyses will be conducted. Because of possible withdrawal before treatment starts, dropouts during the study, failure to fill out questionnaires, or research procedure violations, missing data will occur. Imputation of missing values will be carried out as much as possible to make intention-to-treat analyses feasible. Missing data will be imputed using Imputation and Variance Estimation Software [46].

Prior to the training (T0) we will assess differences between patients and siblings on neurocognitive and psychosocial functioning, using mixed modelling. Subsequently, we will conduct multivariate analysis of variance (MANOVA) to determine the effect of neurofeedback post-treatment (T1) on the primary and secondary outcomes, comparing the patients in the neurofeedback group to the patients in the placebo group. To control for possible differences in neurocognitive functioning prior to the training, T0 data will be included in the model as covariate. Finally, we will use repeated measures analysis for group (neurofeedback and placebo) x time (T0, T1, and six months follow-up, T2) to investigate the changes over time. To examine the possible effects of patient characteristics on the efficacy of the neurofeedback, the following variables will be assessed as covariates in the MANOVA and repeated measures analyses: age at assessment, age at diagnosis, diagnosis, time since diagnosis, and treatment modalities. All analyses will be conducted using SPSS. A *P*-value <0.05 will be considered significant.

## Discussion

This article describes the design of the PRISMA study, a randomized controlled trial investigating the efficacy of neurofeedback in pediatric brain tumor survivors with neurocognitive problems. Although neurocognitive problems in pediatric oncology survivors are reported in numerous studies, empirically validated interventions addressing these deficits are scarce. There is growing evidence for neurofeedback as a valuable treatment in different brain disorders [15,21]. Our study is the first to investigate the efficacy of neurofeedback in pediatric brain tumor survivors using a randomized placebo-controlled double-blind trial, comparing neurofeedback to placebo training. By setting an automatically adjusted threshold of feedback as opposed to a manually adjusted threshold, we enabled blinding the trainers; trainers were not required to monitor the brain activity of the patient during the sessions. Furthermore, we ensure the standardization of the neurofeedback treatment by employing carefully instructed research assistants providing the neurofeedback treatment. At the same time we increased the feasibility for the patients, by administering the training at the patients' home or school. In addition, the effects of neurofeedback on neurocognitive and psychosocial functioning are thoroughly investigated by using well-validated paradigms and psychometrically sound questionnaires administered to patient, caregiver and teacher. Lastly, we have included a control group of healthy siblings, to compare performance of the brain tumor survivors to children without a history of a brain tumor.

The design of PRISMA has some methodological pitfalls to take into account. Because of time factors and the population, this study might be at risk for losing patients during the treatment phase and during follow-up. With five year survival rates of approximately 65%, some patients may relapse [47], or they may discontinue their participation in the study. We increase comparability by employing three different training modules; however, the training might be less effective than an individualized training. The groups receiving each of the three treatment modules are small. Also, the group of brain tumor patients is heterogeneous, e.g. in terms of tumor diagnosis, tumor location, age at diagnosis, treatment, time since diagnosis, and time since end of treatment. It is well documented that these variables play an important role in neurocognitive outcomes. These heterogeneities may be reflected in our results.

In conclusion, if neurofeedback proves to be effective in improving neurocognitive deficits after treatment for a brain tumor, this would be a valuable addition to the currently available effective interventions for this vulnerable group of pediatric brain tumor survivors.

## Competing interest

The authors have no financial relationship or conflicts of interest to disclose.

## Authors’ contribution

MdR, AS, RvM, JG, JO and MG made substantial contributions to conception and design of the study. MdR, AS, RvM, TJ, JG, JO and MG helped drafting the article or revising it critically for important intellectual content. All authors read and approved the final manuscript.

## Pre-publication history

The pre-publication history for this paper can be accessed here:

http://www.biomedcentral.com/1471-2407/12/581/prepub
